# Multi-scale symbolic entropy analysis provides prognostic prediction in patients receiving extracorporeal life support

**DOI:** 10.1186/s13054-014-0548-3

**Published:** 2014-10-24

**Authors:** Yen-Hung Lin, Hui-Chun Huang, Yi-Chung Chang, Chen Lin, Men-Tzung Lo, Li-Yu Daisy Liu, Pi-Ru Tsai, Yih-Sharng Chen, Wen-Je Ko, Yi-Lwun Ho, Ming-Fong Chen, Chung-Kang Peng, Timothy G Buchman

**Affiliations:** Department of Internal Medicine, National Taiwan University Hospital and National Taiwan University College of Medicine, Taipei, Taiwan; Department of Surgery, National Taiwan University Hospital and National Taiwan University College of Medicine, Taipei, Taiwan; Graduate Institute of Communication Engineering, National Taiwan University, Taipei, Taiwan; Department of Agronomy, Biometry Division, National Taiwan University, Taipei, Taiwan; Research Center for Adaptive Data Analysis, National Central University, No. 300, Jhongda Rd, Taoyuan County, 32001 Taiwan; Division of Interdisciplinary Medicine and Biotechnology, Beth Israel Deaconess Medical Center/Harvard Medical School, Boston, Massachusetts USA; Department of Surgery, Emory University School of Medicine, Atlanta, Georgia USA; Division of Cardiology, Department of Internal Medicine, National Taiwan University Hospital, 7 Chung-Shan South Road, Taipei, Taiwan

## Abstract

**Introduction:**

Extracorporeal life support (ECLS) can temporarily support cardiopulmonary function, and is occasionally used in resuscitation. Multi-scale entropy (MSE) derived from heart rate variability (HRV) is a powerful tool in outcome prediction of patients with cardiovascular diseases. Multi-scale symbolic entropy analysis (MSsE), a new method derived from MSE, mitigates the effect of arrhythmia on analysis. The objective is to evaluate the prognostic value of MSsE in patients receiving ECLS. The primary outcome is death or urgent transplantation during the index admission.

**Methods:**

Fifty-seven patients receiving ECLS less than 24 hours and 23 control subjects were enrolled. Digital 24-hour Holter electrocardiograms were recorded and three MSsE parameters (slope 5, Area 6–20, Area 6–40) associated with the multiscale correlation and complexity of heart beat fluctuation were calculated.

**Results:**

Patients receiving ECLS had significantly lower value of slope 5, area 6 to 20, and area 6 to 40 than control subjects. During the follow-up period, 29 patients met primary outcome. Age, slope 5, Area 6 to 20, Area 6 to 40, acute physiology and chronic health evaluation II score, multiple organ dysfunction score (MODS), logistic organ dysfunction score (LODS), and myocardial infarction history were significantly associated with primary outcome. Slope 5 showed the greatest discriminatory power. In a net reclassification improvement model, slope 5 significantly improved the predictive power of LODS; Area 6 to 20 and Area 6 to 40 significantly improved the predictive power in MODS. In an integrated discrimination improvement model, slope 5 added significantly to the prediction power of each clinical parameter. Area 6 to 20 and Area 6 to 40 significantly improved the predictive power in sequential organ failure assessment.

**Conclusions:**

MSsE provides additional prognostic information in patients receiving ECLS.

**Electronic supplementary material:**

The online version of this article (doi:10.1186/s13054-014-0548-3) contains supplementary material, which is available to authorized users.

## Introduction

In recent years, extracorporeal life support (ECLS) has been increasingly used as a life-saving intervention for a variety of critically ill patients [1]. Thus, ECLS has been used as cardiac and/or pulmonary support in various clinical settings, such as fulminant myocarditis, bridge-to-heart transplantation, severe respiratory failure, cardiogenic shock after cardiac surgery, assistance for cardiopulmonary resuscitation (CPR), and septic shock [2-6].

However, the mortality associated with ECLS remains high, and not all critically ill patients will benefit from it [2,4]. The outcome for the ECLS recipient is influenced not only by patient characteristics (disease severity, type of illness, other organ support) [4,7,8], but also by procedural complications related to ECLS [9]. Furthermore, ECLS is a resource-intensive procedure with high cost [10]. Issues about suitability of patients and, more importantly, when to cease ECLS, are particularly important. Therefore, it is important to identify patients who are likely or unlikely to benefit from use of this high-risk, high-cost treatment. Several commonly used scoring systems in the ICU, such as the acute physiology and chronic health evaluation score (APACHE), multiple organ dysfunction score (MODS), sequential organ failure assessment (SOFA), and their successors, have been evaluated in ECLS recipients [8,11]. However, the predictive power among scoring systems varies among studies [8,11-13].

Analysis of the variation of heart rate dynamics, also known as heart rate variability (HRV), is commonly used to assess autonomic function in human studies [14,15] due to its simplicity, noninvasive character, and low cost. Its application to risk stratification of patients with cardiovascular disease has been documented to be independent of conventional clinical parameters [16,17]. In recent years newer methods of calculating and expressing HRV based on nonlinear and non stationary signal modeling have been developed and successfully applied [18-21]. Compared to traditional linear HRV parameters, the nonlinear metrics showed better prediction power for cardiovascular events in several studies [22,23]. One nonlinear method, multiscale entropy (MSE) analysis, was developed to quantify heterogeneous complexity. MSE extends the traditional entropy algorithm to quantify the information richness over multiple time scales that operate in physiological systems [18-20]. In a previous study, MSE provided the best prognostic prediction in patients with heart failure [23]. The utility of MSE extends beyond patients with cardiovascular disease. Thus, MSE also predicts the outcome of patients with severe trauma requiring ICU admission across the diverse spectrum of traumatic injury [24].

Unpredictable yet frequent ectopic beats are common in critical illness. These ectopic beats introduce large artifacts in the calculation of MSE [19]. The conventional approach to using MSE and other measures of HRV is to visually scan, identify and reject those beats, using interpolation procedures to smooth the time series of interbeat intervals. As critically ill patients so frequently have such arrhythmias, visual or computational pre-processing to reject and smooth not only raises the calculation complexity but also can introduce spurious trends or fluctuations into the signals that could limit clinical usefulness. In this paper we introduce a new method, termed multiscale symbolic entropy analysis (MSsE), which is derived from MSE and mitigates the effect of arrhythmia on HRV analysis.

In the current study, we hypothesized that MSsE could yield a prognostic marker in patients receiving ECLS. The aims of this study were 1) to assess the prognostic significance of parameters derived from MSsE; 2) to compare MSsE parameters to conventional clinical parameters; and 3) to evaluate the effect of combining MSsE parameters with conventional clinical parameters.

## Material and methods

### Setting and population

This prospective study was conducted between March 2008 and March 2010 at the National Taiwan University Hospital, which is an ECLS referral center and performs approximately 85 extracorporeal life-support procedures a year [2,6]. Patients were eligible for the present study if they were 18 years or older and had received ECLS for circulatory or respiratory failure that required mechanical support [3]. The decision to use ECLS was made by experienced intensive care specialists or cardiac surgeons. The subjects were enrolled within the first 24 hours after ECLS implantation. Demographic, clinical features and outcomes of patients were recorded. We also recorded the usage of an intra-aortic balloon pump (IABP) or CPR during hospitalization. Catecholamine dose was evaluated by the inotrope equivalent method, that is, calculated as:

$$ \mathrm{mg}/\mathrm{kg}/ \min =\mathrm{dopamine}+\mathrm{dobutamine}+100*\mathrm{epinephrine}+100*\mathrm{norepinephrine}+100*\mathrm{isoproterenol}+15*\mathrm{milrinone}\Big) $$

[11,25].

The APACHE II, SOFA, MODS, logistic organ dysfunction sore (LODS) at ICU admission were calculated according to previous publications [11,26-29]. The primary endpoint was death or urgent cardiac transplantation during the index admission. We followed the patients until discharge or death from the index admission. Healthy volunteers with normal cardiac function evaluated by echocardiography and without cardiovascular disease were enrolled as the control group. The institutional review board of National Taiwan University Hospital approved the present study and informed consent was given by each patient’s family member in the ECLS group due to the unconsciousness of patients and each subject in the control group. Informed consent in the ECLS group was obtained from patients’ family members in the order of spouse, sons or daughters, parents, grandchildren, grandparents, siblings, aunts, uncles, nieces, and nephews.

### Holter recording

All enrolled subjects were placed on standard ambulatory (Holter) electrocardiogram (ECG) recorders for 24-hour recording. The ECG signals were sampled at 250 Hz and stored on an SD card for subsequent offline analysis.

### MSsE calculation

The MSE has proven to be an effective tool in exploring the characteristics of heart rate dynamics and can predict important clinical outcomes [18,23,30,31]. The original MSE comprises two steps: 1) coarse-graining the signals using different time scales; 2) quantifying the degree of irregularity in each coarse-grained time series using sample entropy. However, the major challenge of applying MSE to severe illness patients is the frequent ectopic beats that can reduce the reliability of the MSE results [19]. To address the issue of ectopic beats, we have adopted the intuitive idea that the coarse grained time sequence will be better reconstructed with the median value rather than mean value over non-overlapping windows. In addition, a further step proposed to attenuate the influence from the ectopic beats is to use the sign of the coarse-grained signal to measure its entropy. Dynamics of heart rate after coarse-graining in different scales were therefore denoted as the increase (+) or decrease (−) sign in forming coarse-grained binary sequences. Intuitively, by focusing only on the direction while ignoring the amplitude of the change, the effect of these outliers could be both localized and diminished.

**Figure 1:**
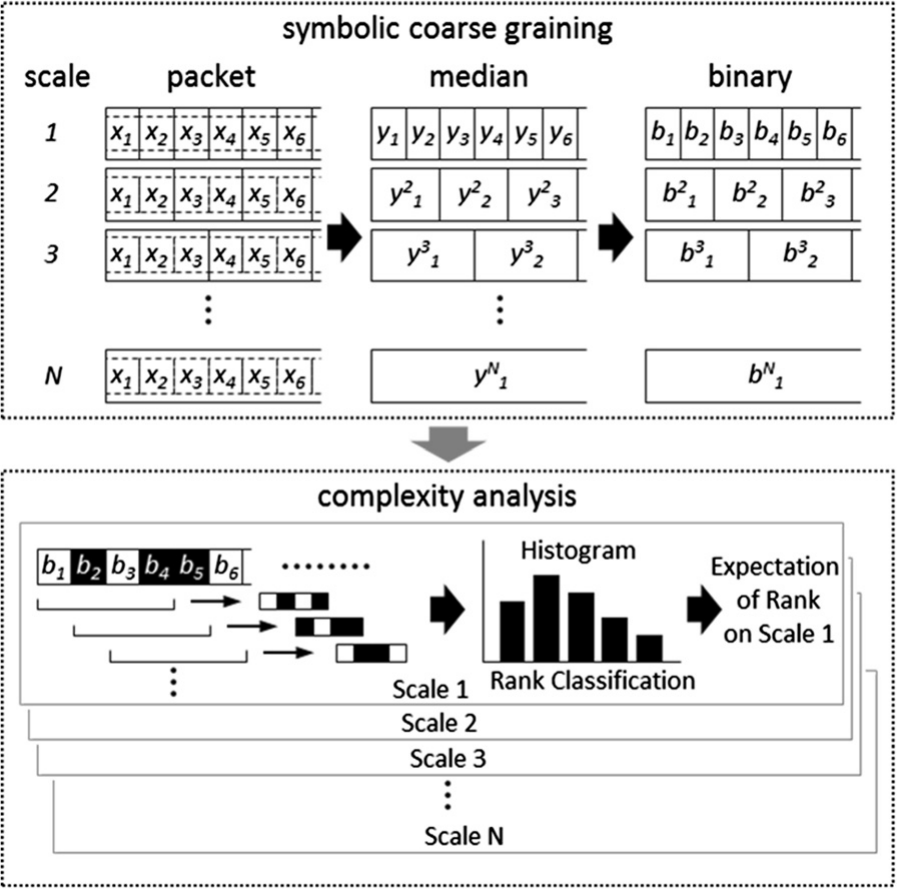
**The first operation of MSsE is coarse graining.** We divide the time series {x_1_, x_2_ … x_M_} into non-overlapping boxes of size *N* (scale "N"). The median of each local box is taken, thus producing a new time series $\left\{{\mathrm{y}}_1^{\mathrm{N}},{\mathrm{y}}_2^{\mathrm{N}}\dots {\mathrm{y}}_{\mathrm{k}}^{\mathrm{N}}\right\}$. The second stage in forming the sign time series is to transform the coarse-grained series into yet another new series by taking the directions in its change. We measure the change against a threshold value and acquire the sign series $\left\{{\mathrm{b}}_1^{\mathrm{N}},{\mathrm{b}}_2^{\mathrm{N}}\dots {\mathrm{b}}_{\mathrm{k}}^{\mathrm{N}}\right\}$, where ${\mathrm{b}}_{\mathrm{i}}^{\mathrm{N}}$ is either +1 or -1, depending on whether the corresponding ${\mathrm{y}}_{\mathrm{i}}^{\mathrm{N}}$ is increasing or decreasing. To quantify the complexity of the sign sequence, we sort all sequences into categories of sub-sets consist of *L* consecutive binary bits (*L*-bit; *L* = 8 in this study). The probability distribution of all patterns of sub-sets is recorded. To avoid over-counting similar patterns, the data sequence of total length L should be divided into multiple m-dimensional vectors; each consists of m consecutive bits {(b_1_, b_2_, … b_m_); (b_2_, b_3_, … b_m +1_); …}. The conditional probability is determined numerically by the ratio of number of each paired vectors which are of exactly same binary codes for dimension "m+1" to the number for the identical vectors of dimension "*m*". By identifying the patterns of the same conditional probability, it allows us to rank the m-bit patterns according to the information they imply (large rank number means lower conditional probability). The expectation value of the rank conceptually indicates the degree of uncertainty.

**Figure 2:**
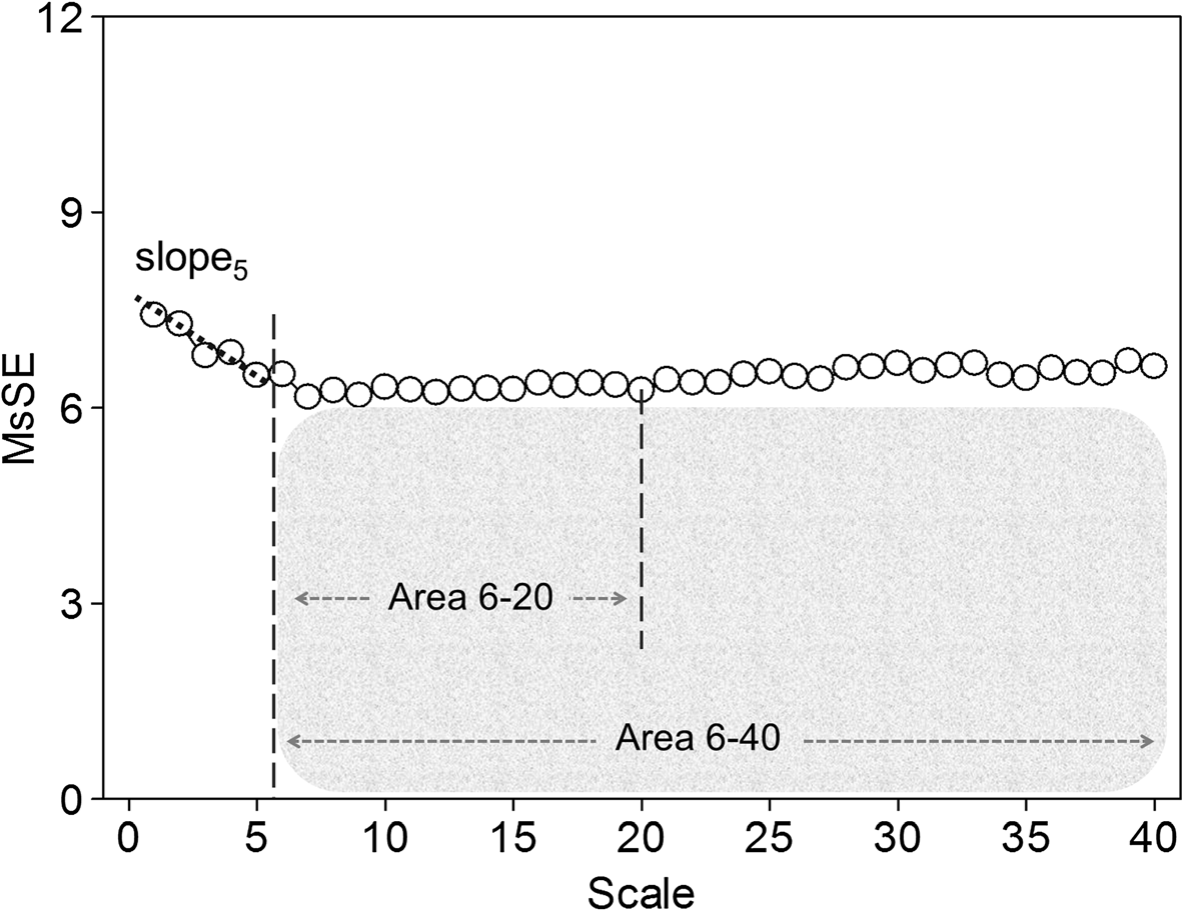
**Quantification of multiscale symbolic entropy (MSsE): summation of the entropy over different scales can quantify the complexity over certain timescales.** However, typical profile of MSsE in extracorporeal life support patients showed a crossover phenomenon around scale 5. Three parameters of the MSsE were assessed: (1) the linear-fitted slope between scales 1 to 5; (2) complexity between intermediate scales (Area 6-20); and (3) the overall complexity (Area 6-40).

In order to compute the entropy of a binary sequence, the sequence should be divided into subsets consisting of *L* consecutive binary bits. The detailed procedure for calculating the entropy of the coarse-grained binary sequences can be found in the caption of Figure 1. We plotted the entropy as a function of scale, which provided the quantitation of structure richness embedded in heartbeat fluctuations over different timescales. This symbolic dynamics approach, now termed multiscale symbolic entropy analysis (MSsE), can provide similar information as the original MSE in quantifying the complexity of the signal while being far less sensitive to the unpredictable yet frequent appearance of ectopic beats. According to previous results that applied MSE to the heartbeat recordings of healthy young subjects [18,19], the sample entropies of heartbeat fluctuations in individuals resembles those of Brownian motions over short timescales (<5 heartbeats) while they behave more like those of pink (1/f) noise over longer timescales. Such a crossover phenomenon is believed to be related to different physiological processes influencing cardiac dynamics over different timescale regions, that is, over short timescales respirations dominantly entrain heartbeat fluctuations (respiratory sinus arrhythmia) via its influence on the parasympathetic nervous system; and over longer timescales (>5 heartbeats), heartbeat fluctuations are influenced by many different physiological functions such as baroreflex and circadian rhythm [32] that lead to complex fluctuation patterns similar to 1/f noise. These findings suggest that we should select Slope_5_ (slope 5), Area_6–20_ (area 6 to 20), and Area_6–40_ (area 6 to 40) as the indices with physiological meaning to profile the MSsE curve (see Figure 2).

We also accessed slope 5 from a shorter time interval of the ECG recording: slope 5 for the first hour and the first 2 hours of ECLS implantation.

### Statistical analysis

Categorical variables were analyzed using the chi-square test or Fisher’s exact test. Continuous variables were represented as mean value ± SD and the normality of those variables was evaluated by using Kolmogorov-Smirnov test. Student’s *t*-test was applied to the between-group comparison. The maximal hazards ratio and independent correlation of variables with event status (mortality or urgent heart transplantation) was determined by Cox regression analysis. Harrell’s *C*-statistics (the probability of concordance for any two randomly chosen subjects) were calculated as a measure of a model’s ability to discriminate between patients meeting and not meeting a primary outcome [33-36]. More specifically:

$$ C=P\left(\;{Z}_i>Zj\;\Big|\;Di=1,\;{D}_j=0\right), $$

where Z_i_, Z_j_ are model-based risks (that is, linear predictors) and D_i_, D_j_ are event indicators for two subjects (1 = patients meeting the primary outcome; 0 = patients not meeting the primary outcome). The receiver-operator characteristic (ROC) curve was determined by the logistic regression model. We performed *C*-statistics to describe discrimination of the baseline model by clinical severity scores and the model that included selected MSsE parameters [34,36,37].

Net reclassification improvement (NRI) and integrated discrimination improvement (IDI) modeling were performed to assess the improvement of the prediction using two different logistic regression models [34], with 0.2 and 0.4 used as cutoff points. All statistical analyses were performed using R software [38], version 2.15.2. Statistical significance was set at *P* <0.05.

## Results

**Figure 3:**
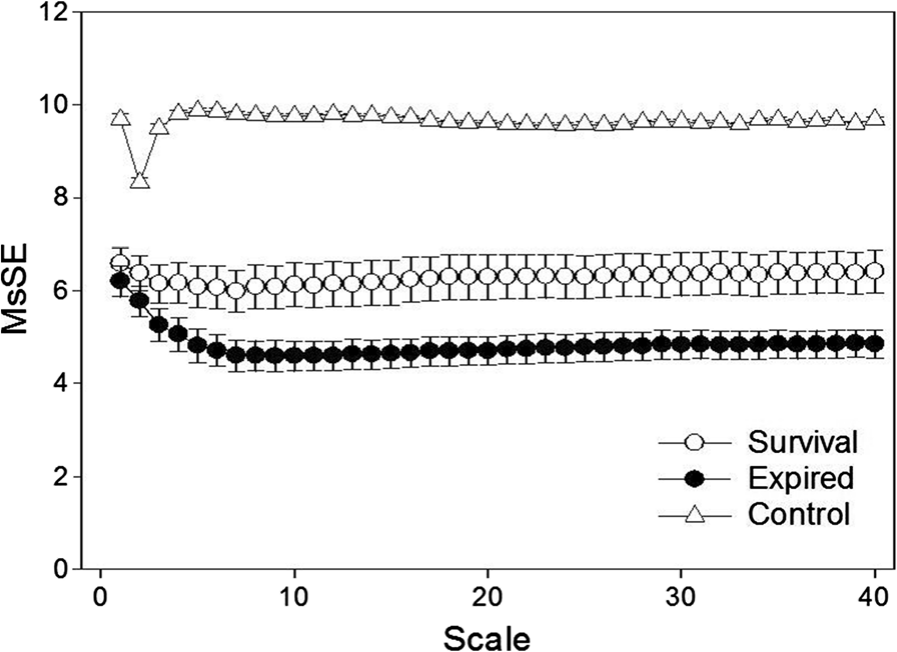
**Multiscale symbolic entropy (MSsE) analysis of heart rate dynamics from control subjects, patients surviving after extracorporeal life support (ECLS), and non-surviving ECLS patients.** The values are represented as mean ± standard error. The MSsE profile of ECLS patients who survived differed from that of non-survivors.

A total of 57 patients (44 male) who were receiving ECLS, and 23 control subjects were enrolled in this study. Of the patients 51 received veno-arterial ECLS and 6 received veno-venous ECLS. The demographic data and data on the MSsE parameters are shown in Table 1. The values of MSsE are also shown in Figure 3. Patients receiving ECLS had significantly lower value of slope 5, area 6 to 20, and area 6 to 40.

**Table 1:** **Clinical and MSsE parameters of all subjects**

	**Control**	**ECLS**	***P*** **-value**
	**(n = 23)**	**(n = 57)**	
Baseline characteristics			
Age, years	58.78 ± 3.16	54.00 ± 18.26	0.061
Sex, male	14 (60.9%)	44 (77.2%)	0.139
Current smoking	1 (4.3%)	18 (31.6%)	0.010
Pre-existing comorbidity			
Diabetes Mellitus	7 (30.4%)	15 (26.3%)	0.709
Hypertension	18 (78.3%)	19 (33.3%)	<0.001
Stroke	1 (4.3%)	2 (3.5%)	0.858
Chronic obstructive pulmonary disease	1 (4.3%)	3 (5.3%)	0.865
End-stage renal disease	0 (0%)	3 (5.3%)	0.262
Coronary artery disease	0 (0%)	13 (22.8%)	0.012
Prior myocardial infarction	0 (0%)	3 (5.3%)	0.262
Heart failure	0 (0%)	17 (29.8%)	0.002
MSsE parameters			
Slope 5	0.19 ± 0.10	−0.24 ± 0.27	<0.001
Area 6 to 20	338.40 ± 6.50	192.04 ± 76.71	<0.001
Area 6 to 40	146.05 ± 2.82	80.77 ± 33.65	<0.001

Results presented as mean ± SD or number (%). ECLS, extracorporeal life support; MSsE, multiscale symbolic entropy.

Among patients receiving ECLS, 35 patients received ECLS for cardiac indications; including 2 patients with acute myocarditis, 9 patients with acute myocardial infarction, 7 patients with intractable heart failure, 6 patients with poor hemodynamic status after cardiac surgery, 8 patients with poor hemodynamic status due to frequent or intractable ventricular arrhythmia, 2 patients with out-of-hospital cardiac arrest and one patient due to massive pulmonary embolism. A total of 22 patients received ECLS for non-cardiac indications; including 2 patients with thyroid storm, 9 patients with acute respiratory distress syndrome, 7 patients with septic shock, one patient with carbon monoxide intoxication, one patient with electrical injury, and two patients with shock due to massive bleeding.

During the index hospitalization, 26 patients died and 3 patients received urgent cardiac transplantation. The other 28 patients were successfully weaned from ECLS and discharged from the hospital alive. In an analysis of baseline characteristics (Table 2), survivors without urgent cardiac transplantation were younger, had longer period of ECLS implantation, lower APACHE score, lower LODS, and lower MODS. With respect to the MSsE parameters (Table 3), survivors without urgent cardiac transplantation had a significantly higher value of slope 5, area 6 to 20, and area 6 to 40.

**Table 2:** **Clinical and laboratory parameters of ECLS recipients**

	**Survivors**	**Non-survivors or patients undergoing heart transplantation**	***P*** **-value**
	**(n = 28)**	**(n = 29)**	
Baseline characteristics			
Age, years	48.07 ± 20.12	59.72 ± 14.39	0.015
Sex, male	22 (78.6%)	22 (75.9%)	0.807
Current smoking	7 (25.0%)	11 (37.9%)	0.294
Pre-existing comorbidity			
Diabetes mellitus	7 (25.0%)	8 (27.6%)	0.825
Hypertension	10 (35.7%)	9 (31.0%)	0.708
Stroke	0 (0%)	2 (6.9%)	0.157
Chronic obstructive pulmonary disease	2 (7.1%)	1 (3.4%)	0.532
End-stage renal disease	2 (7.1%)	1 (3.4%)	0.532
Coronary artery disease	7 (25.0%)	6 (20.7%)	0.698
Prior myocardial infarction	0 (0%)	3 (10.3%)	0.080
Heart failure	6 (21.4%)	11 (37.9%)	0.173
CPR, yes or no	6 (21.4%)	10 (34.5%)	0.273
IABP insertion, yes or no	5 (17.9%)	5 (17.2%)	0.951
Indication of ECLS			0.516
Cardiogenic	16 (57.1%)	19 (65.5%)	
Non-cardiogenic	12 (42.9%)	10 (34.5%)	
Duration of ECLS, day	38.57 ± 31.34	11.69 ± 14.61	<0.001
Inotropic equivalent, μg/kg per minute	70.46 ± 198.44	29.42 ± 27.09	0.275
Clinical parameters			
APACHE score	16.75 ± 7.18	21.83 ± 7.45	0.011
SOFA score	7.71 ± 4.35	9.66 ± 3.42	0.066
LODS	7.61 ± 3.73	10.86 ± 3.59	0.001
MODS	9.61 ± 4.00	12.24 ± 3.59	0.011
Mean arterial pressure, mm Hg	72.38 ± 27.68	63.15 ± 22.10	0.169
Central venous pressure, mmHg	15.17 ± 6.29	16.83 ± 5.93	0.356
Laboratory parameters			
pH	7.29 ± 0.17	7.29 ± 0.20	0.895
PaCO_2_, mmHg	43.76 ± 25.64	47.95 ± 33.32	0.598
HCO_3_, mmol/L	19.48 ± 6.11	20.95 ± 6.76	0.392
Lactate, mmol/L	7.91 ± 5.96	6.87 ± 6.29	0.579
White blood cell count, /μl	13956.40 ± 6512.84	12597.93 ± 82	0.510
Hematocrit, %	34.72 ± 7.20	35.48 ± 7.62	0.711
Blood urea nitrogen, mg/dl	27.23 ± 20.65	38.33 ± 31.63	0.140
Creatinine, mg/dl	1.98 ± 1.75	2.35 ± 2.58	0.551
Total bilirubin, mg/dl	2.31 ± 3.80	2.09 ± 2.22	0.812
Creatinine phosphokinase, U/L	3656.47 ± 8632.73	1064.45 ± 1732.63	0.270
Creatinine phosphokinase-MB	240.82 ± 634.20	87.86 ± 147.04	0.409

Results presented as mean ± SD or number (%). APACHE, acute physiology and chronic health evaluation; CPR, cardiopulmonary resuscitation; LODS, logistic organ dysfunction score, ECLS, extracorporeal life support; IABP, intra-aortic balloon pump; MSsE, multi-scale symbolic entropy; MODS, multiple organ dysfunction score; SOFA, sequential organ failure assessment.

**Table 3:** **MSsE parameters of ECLS recipients**

	**Survivors**	**Non-survivors or patients undergoing heart transplantation**	***P*** **-value**
	**n = 28**	**n = 29**	
Slope 5	−0.12 ± 0.30	−0.35 ± 0.18	0.001
Area 6 to 20	219 ± 85.61	165.69 ± 56.90	0.008
Area 6 to 40	92.41 ± 37.41	69.54 ± 25.48	0.010

ECLS, extracorporeal life support; MSsE, multiscale symbolic entropy.

In Cox regression survival analysis (Table 4), the significant factors were age, prior myocardial infarct (MI) history, APACHE score, LODS, MODS, Slope 5, area 6 to 20, and area 6 to 40. Slope 5 had the largest concordance value among MSsE parameters; the LODS had the largest concordance value among clinical severity scores (Table 5). After combining MSsE parameters with measures of clinical severity, concordance values increased. Among the MSsE parameters, slope 5 added the largest improvement.

**Table 4:** **Cox regression analysis for prediction of the primary endpoint using a single variable in ECLS recipients**

**Variable**	**Exp (B)**	***P*** **-value**	**95% CI for Exp (B)**	
			**Lower**	**Upper**
Age, years	1.026	0.029	1.003	1.050
Sex, male	1.004	0.992	0.428	2.359
Slope 5	0.031	0.003	0.003	0.306
Area 6 to 20	0.992	0.004	0.987	0.997
Area 6 to 40	0.982	0.004	0.971	0.994
Inotropic equivalent, μg/kg per minute	0.998	0.374	0.993	1.003
APACHE score	1.069	0.005	1.020	1.121
SOFA score	1.105	0.054	0.998	1.224
LODS	1.160	0.001	1.060	1.270
MODS	1.176	0.003	1.058	1.307
Diabetes mellitus	1.112	0.799	0.492	2.515
Stroke	3.121	0.124	0.733	13.298
Chronic obstructive pulmonary disease	0.670	0.695	0.090	4.963
End-stage renal disease	0.441	0.423	0.060	3.256
Coronary artery disease history	0.981	0.966	0.399	2.411
Prior myocardial infarction	3.922	0.032	1.125	13.667
Heart failure	1.623	0.219	0.750	3.510
Hypertension	0.981	0.962	0.445	2.161
Current smoking	1.704	0.168	0.799	3.634
ECLS indication, cardiogenic versus non-cardiogenic	1.508	0.301	0.692	3.285

APACHE, acute physiology and chronic health evaluation; CPR, cardiopulmonary resuscitation; LODS, logistic organ dysfunction score, ECLS, extracorporeal life support; IABP, intra-aortic balloon pump; MSsE, multi-scale symbolic entropy; MODS, multiple organ dysfunction score; SOFA, sequential organ failure assessment.

**Table 5:** **Concordance, AUC, NRI, and IDI model among clinical and MSsE parameters**

	**Concordance**	**AUC**	**AUC**	***R*** **square**	**NRI**	**NRI**	**IDI**	**IDI**
			***P*** **-value**			***P*** **-value**		***P*** **-value**
Slope 5	0.731	0.760		0.216				
Area 6 to 20	0.686	0.674		0.151				
Area 6 to 40	0.688	0.670		0.154				
APACHE	0.678	0.706		0.126				
Slope 5	0.740	0.778	0.192	0.258	0.177	0.273	0.133	0.005
Area 6 to 20	0.710	0.741	0.423	0.183	0.143	0.248	0.050	0.094
Area 6 to 40	0.710	0.743	0.429	0.186	0.143	0.248	0.054	0.085
LODS	0.716	0.744		0.168				
Slope 5	0.760	0.804	0.106	0.267	0.389	0.007	0.110	0.009
Area 6 to 20	0.730	0.767	0.300	0.198	0.180	0.161	0.025	0.209
Area 6 to 40	0.730	0.767	0.338	0.198	0.180	0.161	0.026	0.209
MODS	0.689	0.693		0.157				
Slope 5	0.740	0.778	0.102	0.270	0.285	0.087	0.129	0.005
Area 6 to 20	0.740	0.744	0.240	0.224	0.285	0.044	0.059	0.067
Area 6 to 40	0.740	0.748	0.257	0.222	0.285	0.032	0.062	0.059
SOFA	0.598	0.637		0.066				
Slope 5	0.710	0.749	0.086	0.229	0.253	0.101	0.156	0.002
Area 6 to 20	0.690	0.718	0.180	0.173	0.112	0.443	0.089	0.020
Area 6 to 40	0.690	0.720	0.167	0.174	0.181	0.215	0.092	0.018

APACHE, acute physiology and chronic health evaluation; AUC, area under curve; IDI, integrated discrimination improvement; LODS, logistic organ dysfunction score, MSsE, multi-scale symbolic entropy; MODS, multiple organ dysfunction score; NRI, net reclassification improvement; SOFA, sequential organ failure assessment.

**Figure 4:**
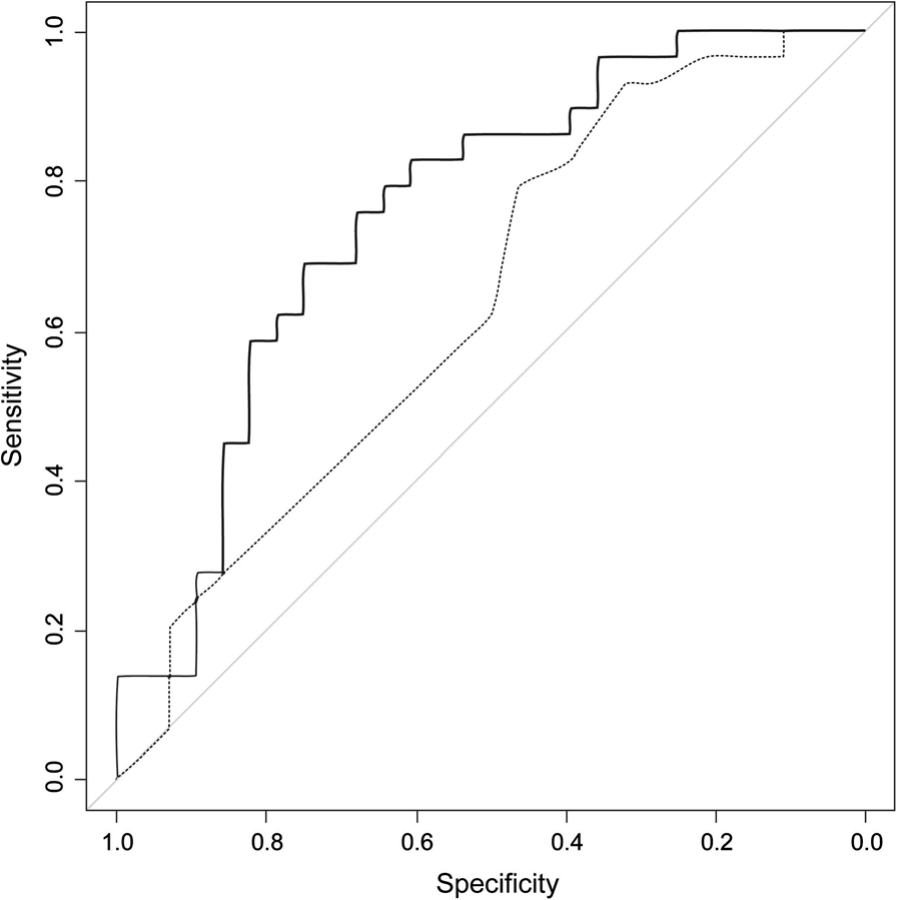
**Receiver-operator characteristic curves for models with (solid line) and without slope 5 (broken line).** The area under the curve (AUC) for sequential organ failure assessment SOFA was 0.637. After adding slope 5, the AUC of the new model improved to 0.749.

In ROC analyses (Table 5), slope 5 had the largest area under the curve (AUC) among MSsE parameters; the LODS had the largest AUC among clinical severity scores. When combining MSsE parameters with measures of clinical severity, AUC improved outcome prediction. Among the MSsE parameters, slope 5 added the largest improvement. For example (see Figure 4), the AUC for SOFA was 0.637. After adding slope 5, the AUC for the new model improved to 0.749.

Although the increase in AUC after adding MSsE parameters on conventional risk scores was not statistically significant, that does not answer the question of whether the addition of MSsE metrics improves the predictive power of conventional risk scores. To answer this question, we considered whether reclassification with the addition of MSsE improved predictions for the individual patients. We used two strategies: NRI and IDI. In general, NRI offers a simple intuitive way of quantifying improvement offered by new markers. As described by Pencina and colleagues, ‘The NRI focuses on reclassification tables constructed separately for participants with and without events, and quantifies the correct movement in categories—“upwards” for events and “downwards” for non-eventsʼ [37]. In the NRI model, slope 5 significantly improved the predictive power of LODS; area 6 to 20 and area 6 to 40 significantly improved the predictive power in MODS. A further improvement on NRI examines all potential cutoff values in the ROC analysis and examines the integral of sensitivity verses the integral of one minus specificity. This is a continuous version of NRI. In IDI model, slope 5 added significantly to the prediction power of each clinical parameter. Area 6 to 20 and area 6 to 40 significantly improved the predictive power in SOFA. The comparison among slope 5 from three time intervals (1 hour, 2 hours, 24 hours) of the ECG recording is shown in Additional file 1.

## Discussion

The major findings of this study were: 1) Among the various clinical and MSsE parameters, slope 5 showed the greatest power in prognostic prediction. 2) MSE parameters (especially slope 5) added the prognostic prediction power in clinical parameters.

The increase in number and severity of critically ill patients worldwide demands that critical care professionals make prudent and objective decisions about the allocation of costly and risky treatments. Such treatments include ECLS, which currently affords survival to only about half the adult patients in which it is initiated. However, due to its high cost [10], issues about the timing to cease ECLS are particularly important. Therefore, it is important to identify patients who are likely to benefit from ECLS. Physiology-based risk-classification tools are therefore needed to support decisions for or against continuous ECLS usage. Although HRV metrics have proved useful in predicting outcomes in some populations, frequent arrhythmias have limited their use in patients with acute and severe critical illness. In this paper, we report the development and application of a new family of HRV metrics based on symbolic entropies that extend the utility of existing nonlinear measures and demonstrate that three members of this new family (slope 5, area 6–20, area 6–40) have the potential to improve conventional clinical risk-stratification tools in determining likelihood of ECLS success.

In our study, the primary outcome rate is quite high (45.6% for mortality, 50.9% for mortality + urgent transplantation). The reasons are probably due to the high disease severity in our participants. Sixteen patients received CPR before ECLS implantation. In our previous experience, the 30-day survival rate of ECLS assisted CPR is 33.9% [2]. Although the mortality rate is still high, however, the outcome of ECLS assisted CPR is much better than conventional CPR [2]. Due to the high mortality and cost of ECLS therapy, effective risk-classification tools are needed to support decisions for or against ECLS initiation and continuation. In a study in Norway, the median estimated cost for the ECMO procedure was 62,545 USD, and the mean estimated total hospital costs is 191,436 USD [10]. In that study, mean duration of an ECLS procedure is 9.5 days (range: 4–23 days). In our study, the average duration of ECLS procedures is 11.69 days in patients met primary outcome. Early risk-classification tools may offer clinician and patient additional insight into their shared decision to use complicated and costly therapies.

In our study, new HRV metrics based on symbolic entropies appeared to enhance risk-classification. These MSsE parameters had good performance in outcome prediction when using alone or combined with clinical parameters. Among the MSsE parameters, slope 5 added the largest improvement. In each clinical parameter, after adding slope 5, the increase of AUC in new model is around 0.06-0.07. The increase exists in all three clinical parameters. There are advantageous features of this tool. First, the cost of measuring MSsE parameters is low and the method can be integrated into ICU data systems. Second, it is measured at bedside, and therefore does not require potentially dangerous transport to an imaging or procedural location. Third, neither radiation nor pharmaceuticals nor additional devices are required.

There are other studies using MSE analysis for acute illness. In trauma patients admitted to ICU, reduced MSE is significantly associated with increasing mortality, and is independent to clinical parameters [39]. In another study, decreased approximate entropy is associated with mortality in trauma patients independent of Glasgow coma score or injury severity score [40]. In a study of combat casualties in an emergency department in Iraq, the MSE index was significantly decreased in patients who required lifesaving intervention compared to those who did not [41]. This evidence shows the potentials of physiology-based risk-classification tools in patients with acute illness. Furthermore, our study demonstrates the additive effects of combination of physiology-based risk-classification tools and clinical parameters.

Interconnectedness of physiological mechanisms is a crucial feature to the output of a network with feedback interactions [42] and demonstrates as multiple time scale correlation for the output fluctuations [43,44]. Multiple time scale correlation traditionally can be assessed by fractal analysis such as detrended fluctuation analysis (DFA) [45-47] i.e., the random time series (no correlation) will exhibit the ~0.5 scaling exponent in the fluctuations vs. time scales for DFA analysis. On the other hand, MSE offer an alternative to verify the existence of multiscale correlation by the slope of the scale function of entropy. Mathematically, a negative slope of MSE analysis indicates the trivial correlation (i.e., simple oscillation, a repetitive pattern of an increase follow by a decrease) or random fluctuations [19]. Therefore, it can be a sign of the loss of feedback regulation. The present study reveals that, in addition to the overall reduction of entropy at different scales (i.e., area 6–20, area 6–40), the patients with death or urgent transplantation show very negative slope (i.e. slope 5) in MSsE analysis. This finding provides preliminary evidence that loss of feedback control could be important features of severe outcome.

In comparison of the outcome predictive power among slope 5 collected from three time interval (from first one hour, from first two hour, and from 24 hours) of ECG recording, generally, the slope 5 from 24 hours have better performance than slope 5 for shorter time ECG recording. However, the slope 5 form one or two hour recording (especially slope 5 form first two hours) still have the ability to predict outcome and also add the performance of clinical parameters. Therefore, parameters form short time ECG recording also have potential for outcome prediction in ECLS patients. Further larger study is need to compare to outcome prediction power of MSsE parameters form short and long time ECG recording in the future.

Our study has several important limitations. It is a single-center study that focuses on the relatively closed population of Taiwan. The study is based on a 24 hour data collection period, an interval that is both long and arbitrary. Our study lumps patients having primarily cardiac disease with patients who have a spectrum of other diseases. This “lumping” convolves and to some extent confuses expression of primary heart pathology with effects of extra-cardiac pathologies upon the heart. The particular entropy metrics that proved useful, while pre-selected for this study, are still somewhat arbitrary. For all of these reasons, our findings must be considered provisional and this study requires replication in another venue to test the reproducibility and consistency of our findings.

These limitations notwithstanding, our study suggests that the addition of metrics derived from non-linear symbolic analysis of heart rate variability improves the ability of conventional risk-stratification tools to create accurate predictions about the outcomes of individual patients within a complicated and very sick population. This is important for two reasons. First, it adds confidence that the “arrhythmia problem in HRV analysis”--the appearance of confounding ectopic beats in critically ill patients--can be addressed objectively and automatically, thus making HRV metrics generally more useful in critically ill patients. Second, it suggests that a more general problem of situation awareness--specifically the ability to make reliable predictions about the outcome of different treatment options--may be improved with the addition of multiscale metrics of physiologic responsiveness to more traditional static indices of physiologic derangement.

## Conclusion

Patients receiving ECLS remain high mortality rate. MSsE offers additional insight into the prognosis of patients considered for ECLS.

## Key messages

- Extracorporeal life support (ECLS) is a resource-intensive procedure with high cost. Issues about suitability of patients and, more importantly, when to cease ECLS are particularly important.
- Multi-scale entropy (MSE) derived from heart rate variability (HRV) is a powerful tool in outcome prediction of patients with cardiovascular diseases. Multi-scale symbolic entropy analysis (MSsE), a new method derived from MSE, mitigates the effect of arrhythmia on analysis.
- In this study, we found several MSsE parameters provide additional prognostic information in patients receiving ECLS. Slope 5 is the best parameter.

## Additional file

Additional file 1:
**Comparison among slope 5 from three time intervals (1 hour, 2 hours, 24 hours).**

## Abbreviations

APACHE: acute physiology and chronic health evaluation
AUC: area under curve
CPR: cardiopulmonary resuscitation
DFA: detrended fluctuation analysis
ECG: electrocardiogram
ECLS: extracorporeal life support
HRV: heart rate variability
IDI: integrated discrimination improvement
LODS: logistic organ dysfunction score
MODS: multiple organ dysfunction score
MSE: multiscale entropy
MSsE: multi-scale symbolic entropy
NRI: net reclassification improvement
ROC: receiver-operator characteristic
SOFA: sequential organ failure assessment
