# Supplementary material for: Multi-scale symbolic entropy analysis provides prognostic prediction in patients receiving extracorporeal life support
Source: Crit Care. 2014 Oct 24;18(5):548. doi: 10.1186/s13054-014-0548-3 (PMC4221713; doi:10.1186/s13054-014-0548-3)
Supplement: Additional file 1: — Comparison among slope 5 from three time intervals (1 hour, 2 hours, 24 hours). [file 13054_2014_548_MOESM1_ESM.docx]

Comparison among slope 5 from three time intervals (1 hour, 2 hours, 24 hours)

During the index hospitalization, 29 patients met primary outcome (26 patients died and 3 patients received urgent cardiac transplantation). The other 28 patients were successfully weaned from ECLS and discharged from the hospital alive. Data of slope 5 from three time intervals (the first 1 hour, 2 hours, and 24 hours) of ECG recording between patients met and unmet primary outcome was shown in Table S1. The slope 5 calculated with 24 hours data shows a greater difference between two groups than slope 5 calculate with first one or first two hours data.

In cox regression survival analysis (Table S2), all three slope 5 indices were significant for outcome prediction. In Table S3, slope 5 calculated with 24 hours record had the largest concordance value and AUC among three slope 5. All three slope 5 indices added the value of concordance value and AUC after combining slope 5 with measures of clinical severity. Among them, slope 5 calculated with 24 hours data had the largest improvement. In NRI model (Table S3), slope 5 calculated with the first 2 hours record significantly improved the predictive power of APACHE and LODS; slope 5 calculated with the first 1 hour record significantly improved the predictive power of LODS and MODS. Slope 5 calculated with 24 hours record significantly improved the predictive power of LODS. In IDI model, both slope 5 calculated with the first 1 and 2 hours data significantly improved the predictive power of APACHE and SOFA. Slope 5 calculated with 24 hours data added significantly to the prediction power of each clinical parameter.

Table S1. Slope 5 from three time intervals in ECLS recipients

|  | Alive  n=28 | Die or Heart transplantation  n=29 | | p-value | |  |
| --- | --- | --- | --- | --- | --- | --- |
| slope 5 1hour | -0.12±0.34 | | -0.28±0.24 | | 0.047 | |
| slope 5 2 hours | -0.13±0.33 | | -0.31±0.22 | | 0.023 | |
| slope 5 24 hours | -0.12±0.30 | | -0.35±0.18 | | 0.001 | |

Abbreviations: ECLS= extracorporeal life support; MSsE= multi-scale symbolic entropy

Table S2. Cox regression analysis for prediction of primary endpoint using slope 5 of three time interval

| Variable | Exp (B) | | p-value | 95％CI for Exp (B)  Lower Upper | |
| --- | --- | --- | --- | --- | --- |
| slope 5 1hour | | 0.249 | 0.036 | 0.068 | 0.911 |
| slope 5 2 hours | | 0.189 | 0.022 | 0.046 | 0.782 |
| slope 5 24 hours | | 0.031 | 0.003 | 0.003 | 0.306 |

Table S3. Concordance, AUC, NRI, and IDI model among clinical parameters and slope 5 of three time interval

|  |  | Concordance | | | AUC | | | AUC  p value | | | R square | | | NRI | | | NRI  p value | | | IDI | | | IDI  p value | | |  |
| --- | --- | --- | --- | --- | --- | --- | --- | --- | --- | --- | --- | --- | --- | --- | --- | --- | --- | --- | --- | --- | --- | --- | --- | --- | --- | --- |
| slope 5 1 hour |  | 0.618 | | 0.642 | | |  | | | 0.078 | | |  | | |  | | |  | | |  | | |  |  |
| slope 5 2 hours |  | 0.607 | | 0.655 | | |  | | | 0.097 | | |  | | |  | | |  | | |  | | |  |  |
| slope 5 24 hours |  | 0.731 | | 0.760 | | |  | | | 0.216 | | |  | | |  | | |  | | |  | | |  |  |
| APACHE |  | 0.678 | | 0.706 | | |  | | | 0.126 | | |  | | |  | | |  | | |  | | |  |  |
|  | slope 5 1 hour | | 0.717 | | | 0.756 | | | 0.344 | | | 0.187 | | | 0.213 | | | 0.096 | | | 0.072 | | | 0.038 | | |
|  | slope 5 2 hours | | 0.710 | | | 0.760 | | | 0.351 | | | 0.197 | | | 0.282 | | | 0.026 | | | 0.085 | | | 0.023 | | |
|  | slope 5 24 hours | | 0.740 | | | 0.778 | | | 0.192 | | | 0.258 | | | 0.177 | | | 0.273 | | | 0.133 | | | 0.005 | | |
| LODS |  | 0.716 | | 0.744 | | |  | | | 0.168 | | |  | | |  | | |  | | |  | | |  |  |
|  | slope 5 1 hour | 0.742 | | 0.776 | | | 0.426 | | | 0.210 | | | 0.283 | | | 0.024 | | | 0.047 | | | 0.084 | | |  |  |
|  | slope 5 2 hours | 0.726 | | 0.770 | | | 0.532 | | | 0.216 | | | 0.319 | | | 0.013 | | | 0.050 | | | 0.076 | | |  |  |
|  | slope 5 24 hours | 0.760 | | 0.804 | | | 0.106 | | | 0.267 | | | 0.389 | | | 0.007 | | | 0.110 | | | 0.009 | | |  |  |
| MODS |  | 0.689 | | 0.693 | | |  | | | 0.157 | | |  | | |  | | |  | | |  | | |  |  |
|  | slope 5 1 hour | 0.719 | | 0.744 | | | 0.188 | | | 0.197 | | | 0.287 | | | 0.024 | | | 0.050 | | | 0.079 | | |  |  |
|  | slope 5 2 hours | 0.712 | | 0.746 | | | 0.192 | | | 0.213 | | | 0.251 | | | 0.063 | | | 0.062 | | | 0.055 | | |  |  |
|  | slope 5 24 hours | 0.740 | | 0.778 | | | 0.102 | | | 0.270 | | | 0.285 | | | 0.087 | | | 0.129 | | | 0.005 | | |  |  |
| SOFA |  | 0.598 | | 0.637 | | |  | | | 0.066 | | |  | | |  | | |  | | |  | | |  |  |
|  | slope 5 1 hour | 0.667 | | 0.725 | | | 0.118 | | | 0.121 | | | 0.108 | | | 0.293 | | | 0.065 | | | 0.043 | | |  |  |
|  | slope 5 2 hours | 0.653 | | 0.718 | | | 0.174 | | | 0.136 | | | 0.110 | | | 0.373 | | | 0.079 | | | 0.028 | | |  |  |
|  | slope 5 24 hours | 0.710 | | 0.749 | | | 0.086 | | | 0.229 | | | 0.253 | | | 0.101 | | | 0.156 | | | 0.002 | | |  |  |
